# Obesity Prevalence and Its Impact on Maternal and Neonatal Outcomes among Pregnant Women: A Retrospective Cross-Sectional Study Design

**DOI:** 10.3390/nursrep14020094

**Published:** 2024-05-17

**Authors:** Wejdan Abdullah A. AlAnnaz, Amel Dawod Kamel Gouda, Faiza Ahmed Abou El-Soud, Mona R Alanazi

**Affiliations:** 1 College of Nursing, King Saud Bin Abdul Aziz University for Health Sciences (KSAU-HS), Riyadh 14611, Saudi Arabia; alannaz17001@ksau-hs.edu.sa (W.A.A.A.); goudaa@ksau-hs.edu.sa (A.D.K.G.); soudf@ksau-hs.edu.sa (F.A.A.E.-S.); 2 King Abdullah International Medical Research Center, Riyadh 11481, Saudi Arabia; 3 Ministry of the National Guard—Health Affairs, Riyadh 11426, Saudi Arabia; 4 Maternal and Newborn Health Nursing, Faculty of Nursing, Cairo University, Giza 11562, Egypt

**Keywords:** obesity, prevalence, pregnancy, maternal and fetal outcomes

## Abstract

Background: The escalating prevalence of obesity in women of reproductive age raises concerns about its impact on maternal and fetal health during pregnancy. This study aimed to thoroughly assess how obesity affects pregnancy and neonatal outcomes among Saudi pregnant women. Methods: In a retrospective cross-sectional study, we analyzed 8426 pregnant women who delivered at King Fahad National Guard Hospital in Riyadh in 2021. Of these, 3416 had obesity, and 341 of them, meeting the inclusion criteria, were selected. Maternal and neonatal outcomes were compiled using a structured questionnaire and extracted from the hospital’s “Best Care” data-based registration system. Results: The findings highlighted that 40.5% of pregnant women were classified as obese, with almost half falling into obesity class II based on BMI. Obesity correlated significantly with adverse maternal outcomes like gestational diabetes and increased rates of cesarean deliveries. Additionally, maternal obesity was linked to unfavorable fetal outcomes, including higher rates of newborn intensive care unit admissions, lower APGAR scores at 1 min, and a greater likelihood of macrosomia. Conclusions: This study underscores the important impact of maternal obesity on both maternal and fetal health during pregnancy. Addressing this high-risk condition demands targeted educational programs for women of reproductive age focusing on BMI control, dietary adjustments, and lifestyle modifications to mitigate obesity-related complications during pregnancy.

## 1. Introduction

Obesity, a multifaceted non-communicable disease, results from a complex interplay of genetic, environmental, hormonal, behavioral, and socioeconomic factors [1,2]. Obesity presents significant threats, amplifying morbidity and mortality rates among affected populations [2]. Dramatically, in the past 20 years, the prevalence of obesity among women of reproductive age has grown significantly as a serious threat to public health over the last few years [3]. Consequently, the World Health Organization (WHO) has nominated obesity as one of the most important threats to human health, defining it as an excessive amount of body fat accumulation and further separating it into three classes according to increasing body mass index (BMI) levels: class I (BMI 30–34.9), class II (BMI 35–39.9), and class III (BMI ≥ 40) [1]. Moreover, obesity is linked to most chronic conditions like type 2 diabetes, hypertension, and cardiovascular disease, collectively contributing to heightened mortality rates [2,4]. Extensive systematic reviews underscore the escalating obesity rates globally, showing an increase in obesity prevalence regardless of geographic location, ethnicity, or socioeconomic status [5]. While obesity rates have risen across demographics, studies indicate a notably higher prevalence among women and older age groups [5].

According to the World Health Organization (WHO) report from 2016, more than 1.9 billion adults were overweight, and one-third of them were classified as obese. This demonstrates a substantial rise in obesity compared to 1975 when the global obesity rate was much lower. The report also highlights that the surge in obesity has been particularly prominent among younger populations, with a specific emphasis on women of childbearing age [6]. The World Health Organization (WHO) has projected that by 2025, the number of adults affected by obesity will continue to increase. It is estimated that 2.7 billion adults will be overweight, with over 1 billion adults classified as obese. Additionally, 177 million adults are expected to be severely affected by obesity [7].

For instance, in the United States, obesity prevalence among women aged 20–39 surged from 28.4% to 34.0% by 2021. Similarly, Sweden reported a 16% increase, Hong Kong saw a 30% rise, and Canada experienced an 82% surge from 1997 to 2009. France witnessed an increase from 8.3% to 15% over the same period [8]. In Europe, the WHO estimates reveal that more than 50% of men and women are overweight, with 23% of women classified as obese [1]. Southeast Asia reports 14% overweight and 3% obese individuals, while in Africa and Southeast Asia, the prevalence of obesity in women is twice that of men [1]. The WHO stated that the overweight and obesity prevalence in KSA is 68.2% (women 69.2% and men 67.5%) and 33.7% (women 39.5% and men 29.5%), respectively [9].

The substantial prevalence of obesity among women of reproductive age carries profound public health implications, particularly concerning adverse effects on pregnancy outcomes. This not only affects women and their offspring but also strains healthcare systems, necessitating heightened healthcare provisions ranging from in vitro fertilization (IVF) to extended antenatal care, cesarean deliveries, and prolonged hospital stays [2,7,9]. Research indicates that the escalating obesity prevalence contributes to heightened incidences of gestational diabetes and macrosomia [10].

Despite the seriousness of this issue, limited research has been undertaken in the Kingdom of Saudi Arabia to assess the prevalence and effects of obesity on pregnancy and neonatal outcomes among pregnant women. This highlights the fundamental importance of the outcomes obtained, offering critical insights into the incidence and consequences among pregnant women. The timely identification of obesity-related complications by nurses and healthcare providers can aid in reducing maternal and birth outcome morbidity and mortality rates, mitigating both short-term and long-term adverse consequences for both mother and fetus. Hence, this study assumes paramount significance in elucidating the effects of obesity on maternal and neonatal outcomes within the context of pregnant women. Therefore, this study aimed to assess the prevalence of obesity among pregnant women and to determine its impact on pregnancy and neonatal outcomes in Saudi Arabia.

## 2. Materials and Methods

### 2.1. Study Design and Setting

A retrospective cross-sectional study design was employed to investigate maternal and fetal outcomes among women with obesity during pregnancy in Saudi Arabia. The study was conducted at the obstetrical and gynecological department within King Fahad National Guard Hospital, King Abdulaziz Medical City, the Ministry of National Guard Health Affairs, Riyadh, Saudi Arabia.

### 2.2. Study Sample

A purposive sampling technique was employed, encompassing all admissions from the commencement of 2021 to the conclusion of the same year. The selection focused on pregnant women exhibiting a high body mass index (BMI) during the third trimester, adhering to specific inclusion and exclusion criteria. These women were identified through medical records at King Fahad National Guard Hospital in Riyadh. The inclusion criteria were defined as follows: (1) residence in Saudi Arabia, (2) age between 18 and 40 years, (3) current pregnancy, (4) carrying a single fetus, and (5) BMI of 30 and above during the third trimester. Exclusion criteria encompassed pregnant women with psychiatric or mental health conditions and those with chronic medical diseases such as diabetes and hypertension. The exclusion of these pregnant women from the study was because of the fact that managing chronic or mental health conditions during pregnancy involves a complex interplay of various factors, including medication, therapy, and support systems. This additional layer of complexity could introduce confounding variables that may complicate the interpretation of the study results.

### 2.3. Recruitment

From the total cohort of 8426 pregnant women who delivered at King Fahad National Guard Hospital in Riyadh in 2021, 3416 were identified as having obesity. Among this subset, 341 pregnant women fulfilled the inclusion criteria and were enrolled in the current study. Data encompassing maternal and fetal clinical outcomes were collected utilizing the hospital’s data registry system, known as “Best Care”, covering the period from the inception to the culmination of 2021. The structured questionnaire employed by the researchers facilitated the compilation of maternal and neonatal outcomes extracted from the “Best Care” data-based registration system within the Obstetrics and Gynecology departments of the hospital.

### 2.4. Tools of Data Collection

A structure data extraction tool was used to collect sociodemographic characteristics, obstetric history, maternal outcomes, and neonatal outcomes. This tool was developed by the authors after an extensive literature review. The questionnaire’s face and content validity were assessed by three expert PhD faculty members in obstetrics and gynecology nursing, ensuring clarity, comprehensiveness, and applicability.

The first part of the questionnaire was demographic data, which included data such as maternal age, weight, height, BMI (BMI: calculated based on the current body weight in kg and height in cm), education status, and residency.

The second part of the questionnaire was obstetrical history, which included data such as gestational age, gravidity, parity, abortion, and mode of previous delivery.

The third part of the questionnaire was the maternal outcomes, which included data such as gestational diabetes, preeclampsia, eclampsia, anemia, premature rupture of membrane, preterm delivery, cesarean section, postpartum complications, intensive care unit admission, and maternal length of stay.

The fourth part of the questionnaire was the neonatal outcomes, which included data such as gestational age, sex of the baby, baby condition, birth weight, 1st minute APGAR score, 5th minute APGAR score, and neonatal intensive care unit admission.

### 2.5. Administrative Approval and Ethical Considerations

Official permissions from the relevant authorities at the study setting were obtained. Ethical approval was secured from the research unit at the College of Nursing at King Saud bin Abdulaziz for Health Sciences and the Institutional Review Board Committee (IRB) with IRB approval number IRB/0913/22 at King Abdullah International Medical Research Center (KAIMRC). Research ethics and hospital protocols were stringently followed to maintain the confidentiality of all patient data.

This study was conducted retrospectively, and due to the nature of the data collection, obtaining individual consent from participants was not feasible. Therefore, a waiver of consent was granted by the Institutional Review Board of KAIMRC for the use of de-identified data. All data analyzed in this study were anonymized to ensure confidentiality and privacy.

### 2.6. Statistical Analysis

Data analysis was performed using SPSS version 22 for Windows. Descriptive statistics, including percentages, means, frequency counts, and standard deviations, were used to describe sample characteristics. The chi-square test was applied for analyzing categorical and ordinal data, while bivariate correlation (Pearson’s test) assessed the association between sample demographic data, obstetrical history, and the effects of obesity among pregnant women.

## 3. Results

A total of 341 pregnant women were included in this study. As shown in Table 1, the mean age of the sample was 30.499 ± 5.236 years. In addition, 56.9% of the sample had a high school education level. The maternal height of the sample was 157.478 ± 7.468. Additionally, the maternal current weight of the sample was 89.771 ± 12.244. Moreover, the maternal pre-pregnancy weight of the sample was 82.094 ± 10.284. Additionally, 73.0% of the sample’s residents were urban. Finally, 44.3% of the sample’s BMI were in obesity class II.

As depicted in Table 1, over 95% of the sample had a gestational age above 36 weeks. Furthermore, 58.4% reported gravidities ranging from 0 to 3. In the same vein, 71.6% had a number of parties totaling between 0 and 3. Additionally, the sample showed that 60.7% had experienced zero abortions and the rest had at least one abortion, and the predominant mode of previous delivery, accounting for 83.6%, was spontaneous vaginal delivery.

The total number of women with normal body weight was 5010 out of 8426 women (59.5%), while the total number of women with obesity was 3416 out of 8426 women (40.5%).

As shown in Table 2, a total of 41.6% of the study sample had gestational diabetes. In addition, 26.7% of the study sample had cesarean section delivery. Additionally, 39.9% of the study samples had postpartum complications. The other complications were less than 10%.

As shown in Table 3, among the study sample (92.1%), the fetal gestational age was term delivery: 37 to 40 weeks. In addition, 7.9% had preterm births. Additionally, 9.7% of the study sample had a low birth weight. Moreover, 26.1% of the study sample were admitted to the NICU. Finally, 17.3% had causes of NICU admission of respiratory distress.

As shown in Table 4, there was a statistically significant relationship between the classes of obesity and seven items of maternal outcome characteristics, including gestational diabetes (chi (ꭓ^2^) test) (*p* = 0.00), anemia (chi (ꭓ^2^) test) (*p* = 0.048), cesarean section (chi (ꭓ^2^) test) (*p* = 0.050), reason for CS (chi (ꭓ^2^) test) (*p* = 0.039), postpartum complications (chi (ꭓ^2^) test) (*p* = 0.00), types of postpartum complications (*p* = 0.001), and maternal length of stay in hospital (Pearson’s test) (*p* = 0.039).

As illustrated in Table 5, there was a statistically significant relationship between classes of obesity categories and two items of fetal outcome characteristics, including birth weight (Pearson’s test) (*p* = 0.00) and first minute APGAR score (Pearson’s test) (*p* = 0.041).

As shown in Table 6, there was a statistically significant relationship between classes of obesity categories and two items of obstetrical variable characteristics, including gravidity (Pearson’s test) (*p* = 0.00) and parity (Pearson’s test) (*p* = 0.00).

## 4. Discussion

This study aimed to investigate maternal and fetal outcomes among women with obesity during pregnancy in Saudi Arabia who were on follow-up at the obstetrical and gynecological department within King Fahad National Guard Hospital, King Abdulaziz Medical City, the Ministry of National Guard Health Affairs, Riyadh, Saudi Arabia. The study conducted broader studies to deepen our understanding of obesity’s prevalence and effects in Saudi Arabia. Obesity is indeed a significant health concern during pregnancy, and the rising prevalence of this condition among pregnant women has been linked to various complications that pose risks to both the mother and the developing fetus, thereby necessitating the need to enhance future interventions and strategies to overcome these outcome complications [11,12].

### 4.1. The Prevalence of Obesity among Pregnant Women

In this retrospective study, the results indicate that the prevalence of obesity among pregnant women is high, and it has significant implications for adverse maternal and neonatal outcomes. The study was conducted based on the data collected from the Department of Obstetrics and Gynecology at King Fahad National Guard Hospital (KAMC) in Riyadh, 2021, where more than one-third of the pregnant women included in the study were classified as obese, specifically 40.5%. This percentage corresponds to a total of 3416 obese pregnant women out of the overall 8426 pregnant women who gave birth that year, which is consistent with the data from European studies. Globally, the prevalence of obesity among pregnant women has risen dramatically in the past 20 years, and recent research studies revealed that the prevalence of obesity among pregnant women in developed countries is higher in developing countries such as the US where it is 42% and Hong Kong where it is 30% (WHO, 2019) [10]. In Finland, 2020, a study found over two-fifths of Finnish pregnant women (41.9%) had obesity [13]. In contrast, a study conducted in Malay by Ying Pang et al., 2016, found that maternal obesity was 21.5%, and in the UK, 14.6% of women with a single pregnancy were obese, as revealed by a study conducted by Barber, Rankin, and Heslehurst, 2017 [14,15]. Meanwhile, another study conducted in Spain showed that the prevalence of obesity in pregnant women ranged from 11.1% in 2012 to 13.4% in 2018 [16]. In addition, one study conducted in Beijing, China, displayed that the prevalence of overweight and obesity in pregnant women was 9.61% [17].

Regarding the Gulf Cooperation Council (GCC) countries, the present study’s findings were in line with the findings of a study conducted by Othman, Himayda, and Shaaban (2018) in Jeddah, Saudi Arabia, where more than one-third of the study sample (39.8%) had obesity during the pregnancy at the Maternity and Children Hospital [18]. In addition, the prevalence of obesity among pregnant women in Oman was 34% (Anita et al., 2018) [19]. On the other hand, the present study’s finding disagrees with a study conducted in Buriada, Saudi Arabia, where 30% of the sample was reported to be obese [20]. Likewise, a study carried out by Fallatah, Alnoury, and Fallatah et al., 2021, showed that 25% of the study sample were obese, which is consistent with the findings of a study conducted in Al-Hassa, Saudi Arabia, where the prevalence of obesity was 29% [21,22]. Comparing the prevalence rates of obesity at international and national levels can be challenging due to several factors, including the divergences in sampling methods, study designs, cultural behaviors, and lifestyle patterns across different countries. These variations make it difficult to directly compare obesity rates between nations and obtain precise estimates.

### 4.2. Effect of Obesity on Maternal Outcome

In this study, the result shows a significant relation between pregnant women with obesity in classes II and III and various complications, and these complications include increased rates of gestational diabetes, cesarean section, and anemia. This finding is in line with recent research studies conducted by Alfadhli (2021), El-Gilany and Hammad (2010), and Kirsten, Shahid, and Sarah (2022), highlighting the relationship between maternal obesity and adverse pregnancy outcomes such as gestational diabetes, cesarean section, and postpartum complications [23,24]. In other studies, the cesarean section rate in the obese group was 42.5%, and it increased to 53.5% among women with obesity in class III and above. These rates are notably higher than the Australian cesarean section rate of 33% reported in 2018 according to AIHW statistics [25].

Additionally, the systematic review and meta-analysis, which analyzed multiple cohort studies, concluded that there is more than a 50% higher risk of cesarean delivery in obese women compared to women of a normal weight. This finding proposes that obesity is indeed a significant risk factor for a cesarean section [26]. Furthermore, a study conducted in Brazil and Australia found that obese women were more likely to have induced labor and require a cesarean section compared to women of a normal weight. This indicated that obesity may increase the likelihood of labor complications that necessitate interventions such as induction and cesarean delivery [27,28]. In addition, a study carried out by Al-Hakmani et al., 2016, concluded that obese mothers are at increased odds of gestational diabetes compared with normal-weight mothers, which could be due to metabolic abnormalities or hormonal changes that can further increase insulin resistance, and gestational diabetes can develop [29]. Similarly, this result is supported by Othman, Himayda, and Shaaban (2021) who stated that the risk of cesarean sections was higher in obese women compared to non-obese women [18]. These results illustrated that there is a higher frequency of cesarean sections, postdated among pregnant obese women, and their pregnancy is considered a high risk, particularly in labor and the postpartum period for both mother and fetus.

Moreover, the current study showed a statistically significant relationship between maternal obesity with postpartum complications, and more than one-third of pregnant women with obesity in classes I and II had postpartum hemorrhage, and more than half of pregnant women with obesity in classes I and II had deep venous thrombosis. This finding is consistent with numerous research studies that have shown that pregnant women who are obese are at an increased risk of experiencing various pregnancy complications. These complications include venous thromboembolism, or thromboembolic disorders, a high risk of postpartum hemorrhage, anemia, gestational diabetes mellitus, gestational hypertension, preeclampsia, the induction of labor, preterm labor, preterm birth, cesarean section, and prolonged pregnancy [30,31,32]. The result of this study accords with Othman, Himayda, and Shaaban (2018) who concluded that there is a statistically significant relationship between maternal obesity and postpartum complications such as deep venous thrombosis and hemorrhage [18]. Moreover, this finding is supported by Shaikh, Robinson, and Teoh (2019), who reported that an increased maternal morbidity is associated with maternal obesity, and there are increased risks of most maternal complications in pregnancy including pre-eclampsia, gestational diabetes, and thromboembolic disorders [33]. The illustration of these complications can arise due to hormonal and physiological changes that occur in a woman’s body to support the growth and development of the fetus. These changes can increase the risk of certain complications, and obesity can further amplify these risks during the postpartum period.

The current study showed that around one-fourth of the study sample stayed in the hospital for more than a week. This finding is consistent with Catalano and Shankar (2017) who showed that obese mothers had an increasing length of stay in the hospital for recovery compared with non-obese mothers which indicates adverse postpartum outcomes associated with maternal obesity [32].

### 4.3. Effect of Obesity Neonatal Outcomes

The findings of this study contribute to exhibiting how different levels of obesity among pregnant women can influence birth weight and fetal growth. This study indicates that pregnant women with obesity in classes II and III have neonates with higher birth weights (macrosomia) compared to those with class I obesity. Additionally, the only statistically significant outcome observed was macrosomia among obese pregnant women. This finding is a line with other findings that revealed the complications associated with neonatal outcomes (macrosomia, stillbirth, low birth weight, and neonatal death). However, the statistically significant outcome observed was macrosomia among obese pregnant women compared to normal-weight women [34,35].

Additionally, the present study observed that more than a quarter of the neonates required immediate admission to the neonatal intensive care unit after delivery due to various health reasons. These reasons include intrauterine growth restriction (IUGR), respiratory distress, congenital defects, acrocyanosis, cyanosis, low birth weight, and meconium aspiration. This finding is supported by several studies that show a direct relationship between maternal body weight and birth weight [36,37]. The higher birth weight, particularly in infants born to obese mothers, is associated with an increased risk of various neonatal complications. These complications can include respiratory distress syndrome, neonatal hypoglycemia (low blood sugar levels), and hyperbilirubinemia (elevated levels of bilirubin in the blood). Infants with these complications may require immediate admission to the neonatal intensive care unit (NICU) for specialized care [23,38,39]. In contrast, a retrospective case-control study at a department of obstetrics in a differentiated perinatal care facility in Portugal reported no significant differences in neonatal intensive care unit admissions for pregnant women with obesity. This variation in the significant differences may be due to many factors that may contribute to differences in NICU admissions, and these factors include the difference in the sample size which could limit its statistical power; variations in healthcare settings, which may have had unique characteristics or practices that influenced the outcomes and access to specialized care and interventions, could contribute to differences in NICU admissions, and different population characteristics also may have had variations in demographic characteristics, socioeconomic factors, or underlying health conditions [40,41].

### 4.4. Limitations and Strengths of the Study

A limitation of this study is its reliance on a cross-sectional retrospective approach, which is adept at establishing associations but falls short in determining causality. Subsequent research endeavors could enhance the study design by opting for longitudinal approaches to track changes over time, offering a more robust foundation for establishing causal relationships between obesity and pregnancy outcomes. Furthermore, this study is crucial as it addresses specific health concerns and provides insights that can significantly impact both maternal and fetal well-being as well as provides the basis for developing evidence-based interventions and guidelines for healthcare professionals to manage and support pregnant women with obesity effectively to ensure that medical practices align with the latest scientific knowledge.

## 5. Conclusions

Maternal obesity significantly impacts both immediate and long-term health outcomes for mothers and newborns, leading to complications such as gestational diabetes, anemia, and an increased likelihood of cesarean section deliveries. Recognizing the complexity of these challenges, our collective efforts in research, education, and clinical practice are crucial for developing effective preventive measures and interventions. To address the unique context of Saudi Arabia, targeted educational programs focusing on BMI control, dietary adjustments, and lifestyle modifications are recommended.

## Figures and Tables

**Table 1:** Sociodemographic and obstetric characteristics of the sample (N = 341).

Variable & Choices	Mean ± SD	Frequency	Percent
Age	30.5 ± 5.24		
Less than 25 years		68	19.9%
26 to 30 years		98	28.7%
31 to 35 years		105	30.8%
36 and above		70	20.5%
Education			
High school		194	56.9%
Bachelor		144	42.2%
Higher studies		3	0.9%
Maternal Height	157.48 ± 7.47		
Maternal Current weight	89.77 ± 12.24		
Maternal pre-pregnancy weight	82.09 ± 10.28		
BMI	35.66 ± 3.69		
Obesity class I (30–34.9)		144	42.2%
Obesity class II (35–39.9)		151	44.3%
Obesity class III (above 40)		46	13.5%
Residency			
Urban		249	73.0%
Rural		92	27.0%
Gestational Age	39.296 ± 1.765		
Less than 30 weeks		0	0.0%
31–35 Weeks		13	3.8%
Above 36 Weeks		328	96.2%
Gravidity	3.352 ± 1.899		
0		0	0.0%
1–3		199	58.4%
4–7		132	38.7%
Above 8		10	2.9%
Parity	2.762 ± 1.573		
0		1	0.3%
1–3		243	71.3%
4–7		96	28.2%
Above 8		1	0.3%
Abortion			
Yes		134	39.3%
No		207	60.7%
Mode of previous delivery			
Nulliparous (has not given birth before)		66	19.3%
Spontaneous vaginal		220	64.5%
Forceps		1	0.3%
Ventose		0	0.0%
Emergency cesarean section		7	2.1%
Elective cesarean section		47	13.8%

**Table 2:** Maternal outcomes among pregnant women with obesity (N = 341).

Variable/Choices		Frequency	Percent
Gestational diabetes	Yes	142	41.6%
	No	199	58.4%
Preeclampsia	Yes	30	8.8%
	No	311	91.2%
Eclampsia	Yes	4	1.2%
	No	337	98.8%
Anemia	Yes	22	6.5%
	No	319	93.5%
Premature rupture of membranes (PROM)	Yes	22	6.5%
	No	319	93.5%
Preterm delivery	Yes	19	5.6%
	No	322	94.4%
Cesarean section (CS)	Yes	91	26.7%
	No	250	73.3%
Reason for (CS)	Macrosomy	2	0.60%
	Breach position	17	5.00%
	Failure of progress	21	6.10%
	Fetal distress	15	4.50%
	High blood pressure	3	0.90%
	Non-reassurance CTG	1	0.30%
	Placenta previa	4	1.20%
	Previous CS	28	8.20%
Postpartum complications	Yes	136	39.9%
	No	205	60.1%
Types of postpartum complications	Post postpartum hemorrhage	48	14.1%
	Infection	4	1.2%
	Adhesion	3	0.9%
	Deep vein thrombosis (DVT)	77	22.6%
	Breastfeeding complications	4	1.2%
	No postpartum complications	205	60.1%
Mode of current delivery	Spontaneous vaginal	186	54.5%
	Forceps	50	14.7%
	Ventose	15	4.4%
	Emergency cesarean section	46	13.5%
	Elective cesarean section	44	12.9%
Intensive care unit (ICU) admission	Yes	22	6.5%
	No	319	93.5%
Mortality	Yes	2	0.6%
	No	339	99.4%
Maternal length of stay in hospital	Less than 5 days	270	79.2%
	6–10 days	58	17.0%
	Above 11 days	13	3.8%
Mean ± SD		4.812 ± 2.179	

**Table 3:** Fetal outcomes among pregnant women with obesity (N = 341).

Variable/Choices		Frequency	Percent
Fetal gestational age	Full-term delivery: 37 to 40 weeks	314	92.1%
	Preterm delivery: 28 to 36 weeks	27	7.9%
Baby condition	Alive	331	97.1%
	Dead	10	2.9%
Birth weight	Low birth weight (>2500 g)	33	9.7%
	Normal weight (from 2500 to 4000 g)	230	67.4%
	Macrocosmic (above 4000 g)	78	22.9%
First minute APGAR score	(0–3 score) needs immediate resuscitation	2	0.6%
	(4–6 score) moderately depressed	17	5.0%
	(7–10 score) normal	322	94.4%
Mean ± SD		8.214 ± 1.134	
5th minute APGAR score	(0–3 score) needs immediate resuscitation	2	0.6%
	(4–6 score) moderately depressed	13	3.8%
	(7–10 score) normal	326	95.6%
Mean ± SD		8.425 ± 1.084	
Neonate intensive care unit (NICU) admission	Yes	89	26.1%
	No	252	73.9%
Causes of NICU admission	Intrauterine growth restriction (IUGR)	5	1.5%
	Respiratory distress	59	17.3%
	Congenital defect	3	0.9%
	Acrocyanosis	1	0.3%
	Cyanosis	18	5.3%
	Low birth weight	1	0.3%
	Meconium aspiration	2	0.6%
	No admission to NICU	252	73.9%

**Table 4:** The association between the classes of obesity and maternal outcomes.

Maternal Outcomes		BMI Classification						Test	*p* Value
		Obesity Class I (144)		Obesity Class II (151)		Obesity Class III (46)			
		Number	%	Number	%	Number	%		
Gestational diabetes	Yes	50	34.7%	61	40.4%	31	67.4%	15.484	0.000
	No	94	65.3%	90	59.6%	15	32.6%		
Preeclampsia	Yes	10	6.9%	15	10.0%	5	10.9%	1.129	0.569
	No	134	93.1%	136	90.0%	41	89.1%		
Eclampsia	Yes	0	0.0%	4	2.7%	0	0.0%	5.073	0.079
	No	143	100.0%	147	97.3%	47	100.0%		
Anemia	Yes	14	9.7%	8	5.3%	0	0.0%	6.058	0.048
	No	130	90.3%	143	94.7%	46	100.0%		
PROM	Yes	8	5.6%	9	6.0%	5	10.9%	1.740	0.419
	No	136	94.4%	142	94.0%	41	89.1%		
Preterm delivery	Yes	10	7.0%	8	5.3%	1	2.3%	1.467	0.480
	No	135	93.0%	143	94.7%	44	97.7%		
Cesarean section (CS)	Yes	30	20.8%	44	29.1%	17	37.0%	5.466	0.050
	No	114	79.2%	107	70.9%	29	63.0%		
Reason for CS	Big baby	0	0.0%	0	0.0%	2	4.3%	27.202	0.039
	Breach position	7	4.9%	8	5.3%	2	4.3%		
	Failure of progress	7	4.9%	9	6.0%	5	10.9%		
	Fetal distress	6	4.2%	7	4.6%	2	4.3%		
	High blood pressure	0	0.0%	3	2.0%	0	0.0%		
	Non-reassurance CTG	0	0.0%	1	0.7%	0	0.0%		
	Placenta previa	3	2.1%	1	0.7%	0	0.0%		
	Previous CS	7	4.9%	15	9.9%	6	13.0%		
	Does not apply	114	79.2%	107	70.9%	29	63.0%		
Postpartum complications	Yes	42	29.2%	65	43.0%	29	63.0%	17.819	0.000
	No	102	70.8%	86	57.0%	17	37.0%		
Types of postpartum complications	Post postpartum hemorrhage	12	8.3%	23	15.2%	13	28.3%	30.655	0.001
	Infection	1	0.7%	3	2.0%	0	0.0%		
	Adhesion	1	0.7%	2	1.3%	0	0.0%		
	Deep vein thrombosis (DVT)	24	16.7%	37	24.5%	16	34.8%		
	Breastfeeding complications	4	2.8%	0	0.0%	0	0.0%		
	Does not apply	102	70.8%	86	57.0%	17	37.0%		
Mode of current delivery	Spontaneous vaginal	86	59.7%	79	52.3%	21	45.7%	9.905	0.272
	Forceps	25	17.4%	19	12.6%	6	13.0%		
	Ventose	4	2.8%	9	6.0%	2	4.3%		
	Emergency cesarean section	13	9.0%	23	15.2%	10	21.7%		
	Elective cesarean section	16	11.1%	21	13.9%	7	15.2%		
ICU admission	Yes	7	4.9%	11	7.3%	4	8.7%	1.161	0.560
	No	137	95.1%	140	92.7%	42	91.3%		
Mortality	Yes	0	0.0%	2	1.3%	0	0.0%	2.531	0.282
	No	144	100.0%	149	98.7%	46	100.0%		
Maternal length of stay in hospital	Less than 5 days	122	84.7%	116	76.8%	32	69.6%	(3.289)	0.039
	6–10 days	19	13.2%	28	18.5%	11	23.9%		
	Above 11 days	3	2.1%	7	4.6%	3	6.5%		
	Mean ± SD	4.500 ± 2.048		4.940 ± 2.186		5.370 ± 2.435			

**Table 5:** The association between the classes of obesity and fetal outcomes.

Fetal Outcomes		BMI Classification						Test	*p* Value
		Obesity Class I (144)		Obesity Class II (151)		Obesity Class III (46)			
		Number	%	Number	%	Number	%		
Neonatal gestational age	Term delivery: 37 to 40 weeks	133	92.4%	137	90.7%	44	95.7%	1.199	0.549
	Preterm delivery: 28 to 36 weeks	11	7.6%	14	9.3%	2	4.3%		
Sex of baby	Male	85	59.0%	69	45.7%	27	58.7%	5.934	0.051
	Female	59	41.0%	82	54.3%	19	41.3%		
Baby condition	Alive	141	97.9%	145	96.0%	45	97.8%	1.033	0.597
	Dead	3	2.1%	6	4.0%	1	2.2%		
Birth weight	Less 2500 g	16	11.1%	14	9.3%	3	6.5%	23.865	0.000
	From 2500 to 4000 g	109	76.2%	100	66.2%	21	45.7%		
	Above 4000 g	19	13.3%	37	24.5%	22	47.8%		
First minute APGAR score	Needs immediate resuscitation	0	0.0%	1	0.7%	1	2.2%	3.003	0.041
	Moderately depressed	6	4.2%	9	6.0%	2	4.3%		
	Normal	138	95.8%	141	93.4%	43	93.5%		
	Mean ± SD	8.361 ± 0.987		8.166 ± 1.224		7.913 ± 1.208			
Fifth minute APGAR score	Needs immediate resuscitation	0	0.0%	1	0.7%	1	2.2%	2.034	0.132
	Moderately depressed	5	3.5%	7	4.6%	1	2.2%		
	Normal	139	96.5%	143	94.7%	44	95.7%		
	Mean ± SD	8.535 ± 0.908		8.397 ± 1.184		8.174 ± 1.217			
NICU admission	Yes	35	24.3%	36	23.8%	18	39.1%	4.689	0.096
	No	109	75.7%	115	76.2%	28	60.9%		
Causes of NICU admission	Intrauterine growth restriction (IUGR)	3	2.1%	2	1.3%	0	0.0%	17.996	0.207
	Respiratory distress	19	13.2%	28	18.5%	12	26.1%		
	Congenital defect	0	0.0%	2	1.3%	1	2.2%		
	Acrocyanosis	1	0.7%	0	0.0%	0	0.0%		
	Cyanosis	10	6.9%	3	2.0%	5	10.9%		
	Low birth weight	1	0.7%	0	0.0%	0	0.0%		
	Meconium aspiration	1	0.7%	1	0.7%	0	0.0%		

**Table 6:** The association between the selected obstetrical variables and classes of obesity.

Obstetrical Variable		BMI Classification						Test	*p* Value
		Obesity Class I (144)		Obesity Class II (151)		Obesity Class III (46)			
		Number	%	Number	%	Number	%		
Gestational age	Less than 30 weeks	0	0.0%	0	0.0%	0	0.0%	2.229	0.109
	31–35 Weeks	4	2.8%	7	4.6%	2	4.3%		
	Above 36 Weeks	140	97.2%	144	95.4%	44	95.7%		
	Mean ± SD	39.528 ± 1.770		39.152 ± 1.769		30.043 ± 1.686			
Gravidity	0–3	101	70.1%	83	55.0%	15	32.6%	11.611	0.000
	4–7	40	27.8%	64	42.4%	28	60.9%		
	Above 8	3	2.1%	4	2.6%	3	6.5%		
	Mean ± SD	2.903 ± 1.779		3.470 ± 1.843		4.370 ± 2.026			
Parity	0–3	115	79.9%	106	70.2%	23	50.0%	10.700	0.000
	4–7	29	20.1%	44	29.1%	23	50.0%		
	Above 8	0	0.0%	1	0.7%	0	0.0%		
	Mean ± SD	2.424 ± 1.480		2.828 ± 1.582		3.609 ± 1.513			
Abortion	0	98	68.1%	86	57.0%	23	50.0%	2.922	0.055
	1–3	46	31.9%	65	43.0%	22	47.8%		
	4–7	0	0.0%	0	0.0%	0	0.0%		
	Above 8	0	0.0%	0	0.0%	1	2.2%		
	Mean ± SD	0.451 ± 0.746		0.0629 ± 0.846		0.739 ± 0.905			
Mode of previous delivery	Nullipara (primigravida)	27	18.8%	24	15.9%	15	32.6%	9.473	0.304
	Spontaneous vaginal	95	66.0%	103	68.2%	22	47.8%		
	Forceps	1	0.7%	0	0.0%	0	0.0%		
	Ventose	0	0.0%	0	0.0%	0	0.0%		
	Emergency cesarean section	3	2.1%	3	2.0%	1	2.2%		
	Elective cesarean section	18	12.5%	21	13.9%	8	17.4%		

## Data Availability

The data presented in this study are available on request from the corresponding author.

## Public Involvement Statement

There was no public involvement in any aspect of this research.

## Guidelines and Standards Statement

This manuscript was drafted against the STROBE for cross-sectional research.
